# Intensification of functional neural control on heartbeat dynamics in subclinical depression

**DOI:** 10.1038/s41398-021-01336-4

**Published:** 2021-04-14

**Authors:** Vincenzo Catrambone, Simone Messerotti Benvenuti, Claudio Gentili, Gaetano Valenza

**Affiliations:** 1 Research Center E. Piaggio & Department of Information Engineering, School of Engineering, University of Pisa, 56126 Pisa, Italy; 2 Department of General Psychology, University of Padua, 35131 Padua, Italy

**Keywords:** Human behaviour, Depression

## Abstract

Subclinical depression (dysphoria) is a common condition that may increase the risk of major depression and leads to impaired quality of life and severe comorbid somatic diseases. Despite its prevalence, specific biological markers are unknown; consequently, the identification of dysphoria currently relies exclusively on subjective clinical scores and structured interviews. Based on recent neurocardiology studies that link brain and cardiovascular disorders, it was hypothesized that multi-system biomarkers of brain–body interplay may effectively characterize dysphoria. Thus, an ad hoc computational technique was developed to quantify the functional bidirectional brain–heart interplay. Accordingly, 32-channel electroencephalographic and heart rate variability series were obtained from 24 young dysphoric adults and 36 healthy controls. All participants were females of a similar age, and results were obtained during a 5-min resting state. The experimental results suggest that a specific feature of dysphoria is linked to an augmented functional central-autonomic control to the heart, which originates from central, frontopolar, and occipital oscillations and acts through cardiovascular sympathovagal activity. These results enable further development of a large set of novel biomarkers for mood disorders based on comprehensive brain–body measurements.

## Introduction

Depression is a severe clinical entity described in the Diagnostic and Statistical Manual of Mental Disorders (DSM), whereas paucisymptomatic, prodromic, and mild forms of depressive conditions have only recently been considered as full-title diagnostic entities^1^. A common condition, variously called minor or subclinical depression, or dysphoria (from Greek: ‘δiσϕoρiα’, literally hard to bear), has gained increasing attention. While it can be considered to be a less severe form of major depression, its effects on a patient’s quality of life and even life expectancy are similar to that of major depression disorder^2,3^. Although not described in the DSM, this mood alteration is characterized by the presence of some depressive symptoms without satisfying all the criteria for major depression: for instance, a widely accepted definition of dysphoria is that it fulfils between two and five symptoms of depression according to the DSM-5^1^ and has a score higher than 12 according to the Beck Depression Inventory-II (BDI–II^4,5^).

Studies investigating clinical and subclinical depression have been primarily focused on brain dynamics of subjects with depressive symptoms, reporting changes in electroencephalographic (EEG) low frequency spectral activity when compared to healthy controls^6,7^, as well as changes in connectivity^8^ and symmetry^7,9,10^ metrics. These results have also been confirmed by neuroimaging investigations^11–13^. However, depression is not just a mental disorder, it is linked to several leading causes of cardiovascular diseases, often referred to as the ‘vascular depression’ hypothesis^14^. Furthermore, cardiovascular dynamics is known to affect significantly the depression risk through direct physical or indirect biological, bodily, or psychosocial changes^15^.

Indeed, cardiovascular diseases are among the most frequent somatic comorbidities of depressive conditions^14,15^. Despite the increasing interest in the literature, only a few studies have specifically assessed whether the clinical interaction between mood and cardiac alterations is bidirectional or if there is a stronger causative relation from mood alterations to heart dynamics, or vice versa. It has been hypothesized that the interplay between depressive and cardiac disorders should be considered as a ‘downward spiral’, in which they strengthen each other^15,16^. Finally, depressive disorders are also associated with a higher risk and worse prognosis of coronary heart disease^17,18^, whereas patients with cardiac diseases have a higher prevalence of depression and depressive symptoms compared with the general population^19,20^.

Similar results have been described for individuals with sub-threshold depression^3,21–23^. Considering the studies cited above, a potential method to investigate the relationship between cardiac and mood alterations is through the autonomic nervous system (ANS). Studies have identified several links between ANS dynamics and depressive symptoms (for a review, refer to ref. ^24^). In particular, a reduced vagal tone has been identified using a linear analysis comparing depressive patients with healthy controls^25^, and a sympathetic hyper-tone was identified in patients with major depression^26^. Moreover, symptoms of depression, such as somatomotor deficits, lower social engagement, and stiff behavioural response, were identified as singularly related to vagal hypo-activity^27^. Further complex and nonlinear methodologies have presented significant differences in people affected by mood disorders compared to healthy controls^25,28^. For instance, a previous study by our group indicated that subclinical depression is associated with a significant increase in heart rate variability (HRV) complexity. This was observed in features extracted from a lagged Poincaré plot and in the first two order moments of the HRV series. Moreover, an inverse trend was observed in parameters from a recurrence quantification analysis with respect to healthy controls^29^. Interestingly, in ref. ^30^ authors hypothesized that the neural control over ANS is higher in patients with mood disorders than healthies, because of the increased risk of cardiovascular diseases. Nevertheless, more general clinical evidence indicates that a bidirectional relationship exists between a patient’s mood and cardiovascular dynamics and function^31^, and that depressive conditions are associated with an alteration of the ANS modulating cardiac activity. Thus, little is known regarding the directionality of such alterations. However, while easy to monitor, the use of cardiac biomarkers is challenging, particularly because of specificity issues in human cardiovascular pathophysiology. In fact, different severity of a specific disease may exhibit a clear correlation with cardiac biomarkers, but many other psychophysiological stressors (e.g. emotional elicitations, cognitive load, autonomic maneuverers), may exhibit similar variations. To overcome this limitation as a complement to the approach of studying the relationship linking depressed mood and heart dynamics, the associations between ANS and central nervous system (CNS) should be considered, which is usually referred to as Brain–Heart Interplay (BHI).

**Fig. 1:**
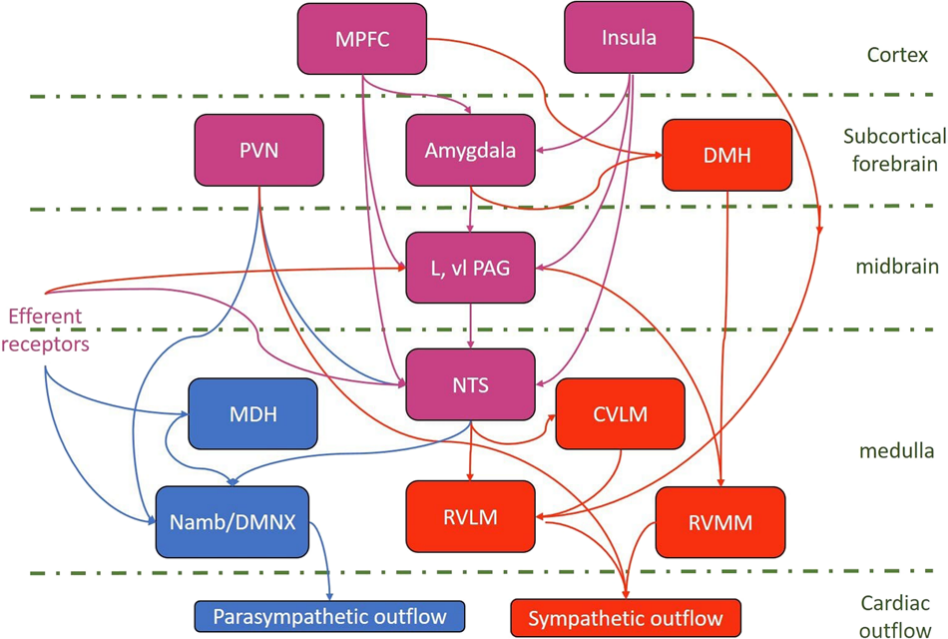
CAN scheme. Schematic diagram showing the major central pathways regulating cardiac sympathetic (red) and parasympathetic (blue) outflows. Common pathways are indicated in purple. No distinction is made between the excitatory and inhibitory connections. DMNX dorsal motor nucleus of the vagus nerve, l lateral, MDH medullary dorsal horn of the trigeminal nucleus, MPFC medial prefrontal cortex, NAmb nucleus ambigus, NTS nucleus of the tractus solitarius, PAG periaqueductal grey matter, PVN hypothalamic paraventricular nucleus, vl ventrolateral, CVLM caudal ventrolateral medulla, DMH dorsomedial hypothalamus, RVLM rostral ventrolateral medulla, RVMM rostral ventromedial medulla (for more abbreviations, refer to text).

Indeed, more deeply in the CNS, the complexities of the functionalities and brain regions involved in autonomic control over the heart have been identified and defined as the central autonomic network (CAN)^32–35^. The CAN consists of several components comprising sympathetic and parasympathetic connections to the CNS, as summarized in Fig. 1, and is involved in acute and chronic stress responses^14^. Even though several activities of CAN regions have been found significantly altered in depression^13^, the role of CAN in depressive mood alterations is still under debate. From a methodological viewpoint, the time-varying estimation of functional BHI can be achieved by employing several techniques^36^, most of them based on EEG recordings, which allows for a good time resolution and an acceptable spatial resolution onto the scalp. A classical approach is to look for EEG synchronizations time-locked to the heartbeats, that is, the heartbeat-evoked potentials (HEP) analysis^37^, which is known as an index of the directional interaction going from heart to brain^38^. Most of the other BHI estimation methods exploit information-transfer coupling quantifiers adapted to the BHI application, such as the maximal information coefficient^39^, Granger causality index^40^, transfer entropy^41^, or convergent cross-mapping^42^. Recently, an ad hoc synthetic data generation (SDG) model was presented^43,44^ that quantifies the directional time-varying interplay among different EEG and HRV frequency bands in both directions and in a physiologically inspired fashion.

In light of the above, and the information found in refs. ^5,29,45^, with the aim of investigating the functional directional BHI in individuals with dysphoria, in this study the SDG and HEP estimation methods were applied during a short-term resting state, and the results were compared with analogous measurements from healthy volunteers.

## Materials and methods

### Participants’ enrolment and study design

Sixty undergraduate female students from the University of Padua (with an average age of 21.89 ± 2.06 years) volunteered to participate in this study. Sample size was determined based on previous studies. They gave informed consent to the protocol, which was previously approved by the Department of General Psychology Ethical Committee, University of Padua (Italy). Participants were not taking antidepressants or medicines that could affect ANS or CNS activity. Moreover, they did not have alcohol use disorders or diseases related to neurological or cardiovascular systems. Moreover, the researchers asked the participants not to consume coffee from 2 h before the experimental acquisition.

During the 5-min resting state, the subjects were seated on a comfortable chair in a silent, soundproof, and dark environment. To minimise artefacts, participants were asked not to move or talk throughout the protocol.

The experimental acquisition comprised EEG and ECG recordings. The EEG was gathered from a 32-channel Electro-Cap (Electrocap, Inc.) with tin electrodes placed following the 10–20 standard. Impedance electrodes were maintained below 5 kΩ. The EEG was amplified using a Neuroscan Synamps (El Paso, Texas, USA), which was filtered online in the range of 0.1–70 *Hz*, sampled and digitised at 500 Hz (AD converter at 16 bits, with an accuracy of 0.034 μV/bit), and finally saved on a Pentium II computer. The ECG was acquired using Ag/AgCl shallow electrodes placed on the subject’s chest, following a modified lead II configuration. The recording was amplified (with a gain of 150), filtered (between 0.3 and 100 Hz), sampled at 500 Hz (16-bit A/D converter; with a resolution of 0.559 μV/LSB). Participants were asked to fulfil the following psychometric tests before beginning the experiment:

- The BDI–II test^4,46^ to investigate the presence of possible depressive symptoms. This questionnaire is acknowledged as a valid self-report test that evaluates the severity level of depression over a preceding period of 2 weeks. Responses are on a 4-point (0–3) Likert scale, and scores may be between 0 and 63; a higher score indicates more severe depressive symptoms. A score above 12 in the Italian variant is considered as an indicator of depression problems in the Italian population^46^.
- The State and Trait Anxiety Inventory (STAI) test, which consists of two independent questionnaires (STAI-Y1 for state anxiety and STAI-Y2 for trait anxiety), both consisting of 20 multiple-choice items^47^. The idea behind the test is the conceptual distinction between ‘state’ and ‘trait’ anxiety; with these questionnaires, a physician is able to distinguish anxiety as a transitory state from anxiety as a relatively stable personality trait.
- The Emotion Regulation Questionnaire (ERQ)^48^, which is considered to quantify a person’s inclination in regulating her/his emotions through expressive suppression and cognitive reappraisal. The ERQ test consists of a 10-item scale, and each item has a 7-point Likert-like scale, ranging from 1 (strongly disagree) to 7 (strongly agree).

Moreover, participants with a BDI–II score that exceeded a threshold of 12 were submitted to the mood episode module (module A) of the SCID-I^49^ by an expert psychologist. This proved the presence of dysphoria and excluded those subjects who met the criteria for a diagnosis of mood disorders, dysthymia, or major depression.

Participants’ age, health status, smoking habits, and regular alcohol use were then investigated through a personal interview that the subjects were asked to attend after completing psychometric tests.

Participants were part of a broader group of 224 University of Padua undergraduate students who had completed an online version of the BDI–II. Participants were enrolled in the study only if they achieved a BDI–II score ≤8 or ≥12. Subjects who received a BDI–II test score ≥12, manifesting for at least 2 weeks between two and four depressive symptoms, were assigned to the dysphoric group. Individuals who achieved a BDI–II score ≤8 (belonging to the 53rd percentile), and did not show any depressive symptoms, as defined by the SCID-I, were enrolled as healthy controls. Finally, the non-dysphoric group comprised 36 subjects, whereas the dysphoric group comprised 24 subjects. Table 1 presents the primary general characteristics of both groups.

**Table 1:** Participants’ characteristics represented by their Median value (25th, 75th, percentile) of each group.

Variables	Whole group	Controls (36)	Dysphoric (24)
Age (y)	22 (20, 24)	23 (20, 26)	21 (20, 22.5)
Education (y)	16 (14, 17)	16 (15, 17)	15 (14, 17)
STAI-Y1	33 (30, 36)	31.5 (29.5, 34.5)	34 (32, 40)
STAI-Y2	39.5 (33.5, 52)	35 (31, 39)	52.5 (49, 61.5)
BDI–II	7 (2, 13.5)	2.5 (1, 5.5)	14 (12.5, 20.5)
ERQ-R	31 (25.5, 33.5)	31 (29, 35)	29.5 (23, 32.5)
ERQ-S	13 (8.5, 17)	12 (7.5, 15.5)	14.5 (11, 17)

ERQ-R represents ERQ-Reappraisal; ERQ-S represents ERQ Suppression.

The present study was conducted within an extensive research project, and most of the participants’ data have also been described in the previous publications^29,50,51^. Data were collected between February and May 2014, and a novel approach to data analysis has been applied in the present study.

### Estimation of functional brain–heart interplay

The EEG recordings were pre-processed using the so-called HAPPE pipeline, proposed by ref. ^52^. To summarise, the pipeline extracts the average log power normalised joint probability, from 1 to 70 Hz, and the electrodes that are external to the 1% tails of the distribution are rejected as bad channels. The rejected electrodes are recovered through a spherical interpolation algorithm employing neighbouring channels. A multitaper regression algorithm was employed to filter out spectral components below 1 Hz, the main electrical frequency noise at 50 Hz, and its first harmonic^52^. A wavelet-enhanced independent component analysis (ICA)-based algorithm detected and rejected muscular and ocular artefacts and discontinuities. An additional fast-ICA algorithm was applied, and the derived components were input to a machine learning algorithm that recognises artefact components^52^. Finally, a re-referencing procedure was applied, which employed the time-varying average from all electrodes^53^.

For the ECG series, a Pan–Tompkins algorithm^54^ detected R-peak events. Possible physiological or algorithmic artefacts in the RR-series (such as ectopic beats or peak misdetections) were detected and corrected using point-process statistics, which included a log-likelihood prediction^55^. For more details on a heartbeat dynamics analysis performed on this dataset, please refer to ref. ^29^. A visual inspection analysis ensured that the quality of the EEG and RR series was retained for further analyses.

#### Spectral analysis

For the EEG, the power spectral density (PSD) was estimated using the Welch method, employing a Hamming window of 500 samples (1 s) with a 75% overlap. The PSD was integrated in the five classical EEG frequency bands: δ ∈ [1−4 Hz), θ ∈ [4−8 Hz),

$${\upalpha} \in \left[ {8 - 12\,{\mathrm{Hz}}} \right),\,{\upbeta} \in \left[ {12 - 30\,{\mathrm{Hz}}} \right),\,{\mathrm{and}}\,\gamma \in \left[ {30 - 70\,{\mathrm{Hz}}} \right].$$

For the HRV, the smoothed pseudo-Wigner–Ville distribution method (SPWVD) was employed^56^. It estimates the PSD with a relatively low variance, and it has independent control of filtering in the temporal and frequency domains^57^.

#### Quantification of functional brain–heart interplay

The functional directional BHI was estimated using the SDG model, designed in ref. ^43^. Therefore, the EEG series is modelled using the oscillators model proposed by ref. ^58^, in which amplitudes (i.e., *a*_*j*_ (*t*_*n*_)) are shaped using an exogenous autoregressive model of the first order:

1 $${\mathrm{EEG}}\left( {t_n} \right) = \mathop {\sum}\nolimits_{j = 1}^K {a_j\left( {t_n} \right){\mathrm{sin}}\left( {{\upomega }}_jt_n + \phi _j \right)}$$

2 $$a_j\left( {t_n} \right) = \eta _ja_j\left( {t_{n - 1}} \right) + \xi _j^\prime \left( {t_{n - 1}} \right) + \Psi _j\left( {t_{n - 1}|P_{B_C}\left( {t_{n - 1}} \right),C_{B_C \to j}\left( {t_{n - 1}} \right)} \right)$$

where $j \equiv B$, $B_C \in {\mathrm{LF}} = \left[ {0.04,0.15} \right]{\mathrm{Hz}},{\mathrm{HF}} = \left[ {0.15,0.4} \right]{\mathrm{Hz}},{\mathrm{HT}} = \left[ {0.04,0.4} \right]{\mathrm{Hz}}$, and $C_{B_C \to j} \equiv C_{{\mathrm{Heart}} \to {\mathrm{Brain}}}$. Following this definition, the model formulates the heart-to-brain interplay as:

3 $${{\Psi }}_j\left( {t_{n - 1}} \right) = C_{B_C \to j}\left( {t_{n - 1}} \right) \times P_{B_C}\left( {t_{n - 1}{\mathrm{|}}{\cal{H}}_{t^{\prime}}^{\cal{C}}} \right)$$

with ${\cal{H}}_{t^{\prime}}^{\cal{C}}$ and $P_{B_C}\left( {t_{n - 1}} \right)$ representing past heartbeat dynamics and the heartbeat PSD, respectively. The model shapes the RR series with an integral pulse frequency modulation model, as in:^59^

4 $${\mathrm{RR}}\left( t \right) = \mathop {\sum}\limits_{k = 1}^N {\delta ^\prime \left( {t - t_k} \right)}$$

where *δ*′ denotes a Dirac delta function, *t* is the time in the continuous domain, and *t*_*k*_ is the time of the *k*^*th*^ heartbeat occurrence determined from:

5 $$1 = {\int_{t_k}^{t_{k + 1}}} {\left[ {{\mathrm{HR}} + m\left( t \right)} \right]dt}$$

where HR represents the mean heart rate, expressed in *Hz*. The autonomic activity function, represented as *m(t)*, is formulated as:

6 $$m\left( {t_n} \right) = C_{{\rm{LF}}}\left( {t_n} \right)\sin \left( {\omega _{{\rm{LF}}}t_n} \right) + C_{{\rm{HF}}}\left( {t_n} \right)\sin \left( {\omega _{{\rm{HF}}}t_n} \right)$$

7 $$C_{{\rm{LF}}}\left( {t_n} \right) = C_s^\prime + \Psi _{{\rm{LF}}}\left( {t_{n - 1}|P_j\left( {t_{n - 1}} \right),\;C_{j \to {\rm{LF}}}\left( {t_{n - 1}} \right)} \right)$$

8 $$C_{{\rm{HF}}}\left( {t_n} \right) = C_p^\prime + \Psi _{{\rm{HF}}}\left( {t_{n - 1}|P_j\left( {t_{n - 1}} \right),\,C_{j \to {\rm{HF}}}\left( {t_{n - 1}} \right)} \right)$$

with $C_{j \to B_C} \equiv C_{{\rm{Brain}} \to {\rm{Heart}}}$. Thus, the from-brain-to-heart interplay function $\Psi _{B_C}\left( {t_{n - 1}} \right)$ is defined as:

9 $$\Psi _{B_C}\left( {t_{n - 1}} \right) = C_{j \to B_C}\left( {t_{n - 1}} \right) \times {\rm{PSD}}_{{\rm{EEG}}_j}\left( {t_{n - 1}|{\cal{H}}_{t^{\prime \prime} }^{\cal{B}}} \right)$$

where $B_C \in \{{\rm{HF}},{\rm{LF}}\}$, as in Eqs. (7) and (8); ${\cal{H}}_{t^{\prime \prime} }^{\cal{B}}$ and ${\rm{PSD}}_{{\rm{EEG}}_j}\left( {t_{n - 1}} \right)$, represent the brain activity history and the related time-frequency, respectively. The time-varying heart-to-brain and brain-to-heart interactions are quantified through the directional BHI biomarkers as $C_{B_C \to j}\left( t \right)$ and $C_{j \to B_C}\left( t \right)$, respectively, which have the same time resolution as the input PSDs. Following this model, both the electrophysiological dynamics (i.e., EEG and HRV series) are mutually dependent, and their interaction is modulated by the introduced coupling terms. In summary, a positive $C_{\delta \to {\rm{LF}}}\left( {t_k} \right)$ indicates that the EEG-δ band, at time *t*_*k*_, leads to a linearly proportional increase (i.e. exerting a positive influence) in the HRV-low frequency (LF) band PSD time course. Employing the inverse model formulation, described in detail in ref. ^43^, leads to the derivation of an entire family of BHI biomarkers.

Through this framework, the directional BHI indices listed in Table 2 were derived. To implement the model, an easy-to-use MATLAB (Mathworks) implementation was exploited, which is freely available at ref. ^60^.

**Table 2:** BHI indices extracted through the model.

Index	From	Band	To	Band
$C_{{\rm{Brain}}_j \to {\rm{Heart}}_{B_C}}$	Brain	δ, θ, α, β, γ	Heart	LF, HF
$C_{Heart_{B_C} \to Brain_j}$	Heart	LF, HF, HT	Brain	δ, θ, α, β, γ

In summary, the model quantifies the functional from-brain-to-heart directional interplay as well as the from-heart-to-brain directional interplay throughout the EEG oscillations in different frequency bands (i.e. δ, θ, α, β, and γ) and HRV power in the low-frequency and high-frequency (HF) bands. Following recent evidence on autonomic dynamics, in this study, HRV-LF power was considered as a marker of sympathovagal activity, and the HF power a vagal activity marker^61–64^. Furthermore, the functional interplay was investigated in the from-heart-to-brain direction, originating from the whole HRV spectrum, which is associated with the LF+HF power, namely HT (i.e. [0.04–0.4] Hz).

Intra-subject time-varying BHI estimates were condensed using the median value, and between-group statistical differences are shown as *p*-value topographic maps from a non-parametric Mann–Whitney test for independent samples. The statistical significance threshold was chosen to be α = 0.05, and *p*-values were adjusted for multiple comparisons through permutation tests, with 1000 permutations. A cluster-mass permutation correction was applied to assess the physiological plausibility of the results^65^. Preliminary results of this study were published in^45^.

#### Heartbeat-evoked potentials analysis

To investigate further the causal activity originating from heartbeat dynamics in the brain, a heartbeat-evoked potentials (HEP) analysis was also performed. The HEP technique was proposed in ref. ^37^, and the actual existence of such potentials was documented^66,67^, particularly in the frontal and central cortex^68^ and in the somatosensory cortex^66^. Note that such areas are consistent with CAN regions^32,33,35^. The HEP calculation was performed on a positive EEG potential, bound in the range of [250–500] ms, after the ventricular contraction (i.e. the R-peak timing of the ECG)^68^. Group-wise grand-average estimates from all recordings of such potentials were used to investigate differences between the dysphoric and healthy control groups.

## Experimental results

The experimental results show functional BHI changes associated with dysphoric individuals in the short-term resting state with respect to the healthy controls. To this end, the BHI synthetic data generation computational model (SDG)^43^ was applied using the EEG and HRV series.

**Fig. 2:**
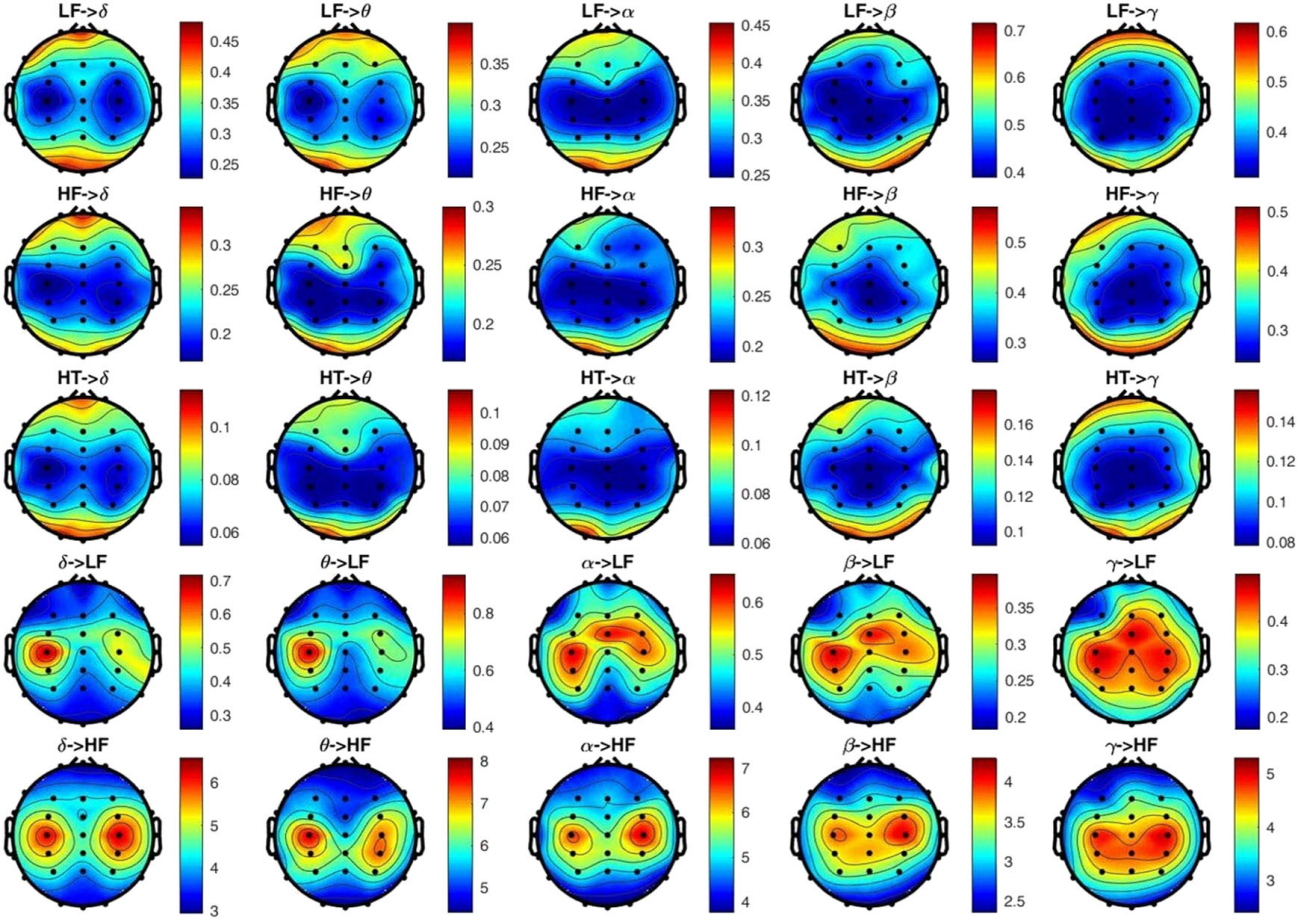
Topographic distribution of the extracted BHI indexes in healthy participants during a resting state. BHI indexed are explained in Table 2. (arbitrary units).

**Fig. 3:**
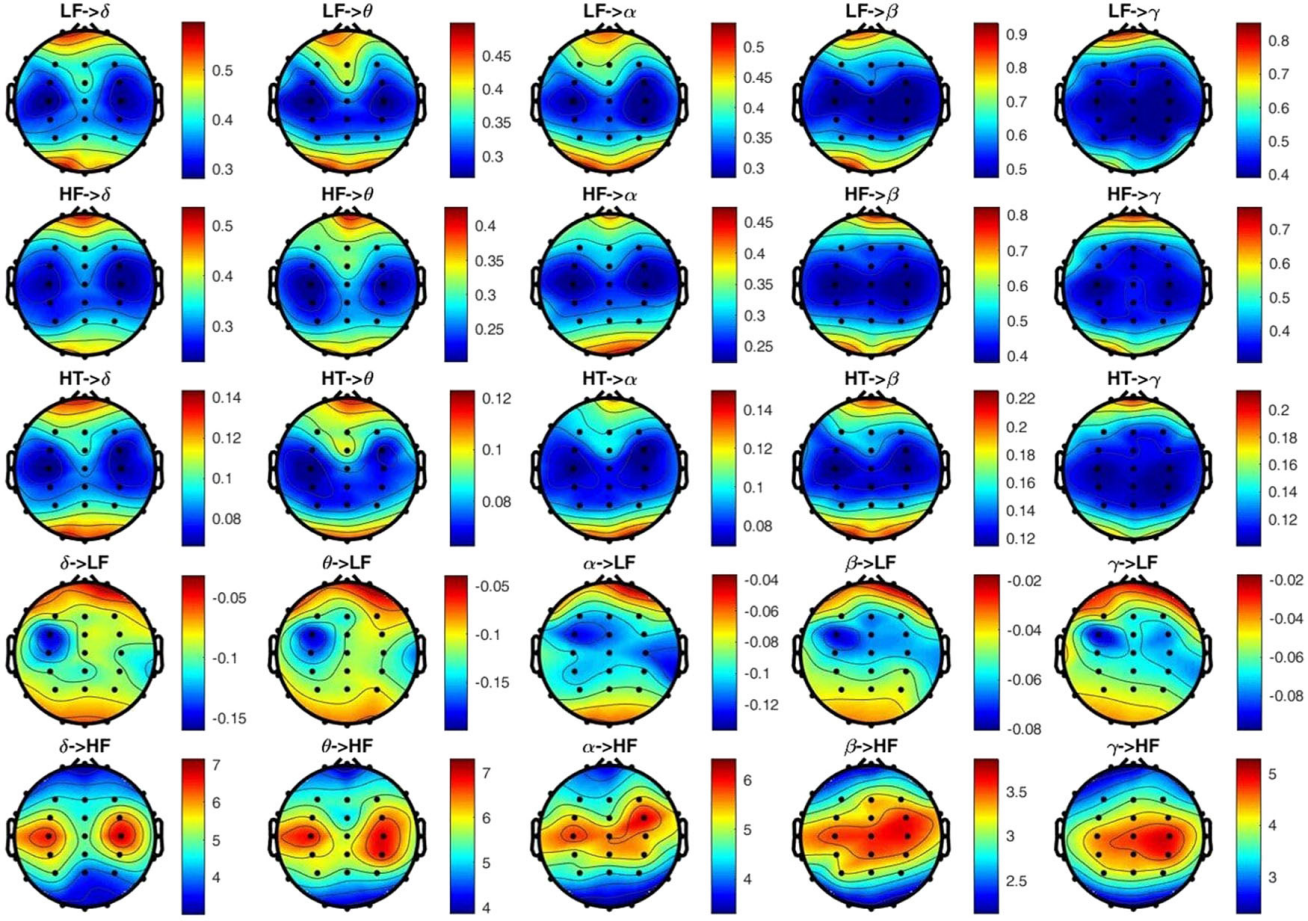
Topographic distribution of the extracted BHI indexes in participants with dysphoria during a resting state. BHI indexes are explained in Table 2. (arbitrary units).

Figures 2 and 3 show group-wise statistics (median) for BHI biomarkers in the dysphoric group and the control group, respectively. Interestingly, all the maps related to the results that involve the from-heart-to-brain interaction, in both groups, exhibit a gradient from the central region of the scalp to the peripheral regions on the medial-axis. This indicates that BHI values in the central and temporal lobes are lower than those in the frontal and occipital areas. For the opposite BHI direction, i.e. from-brain-to-heart, it can be observed that an inverse gradient is present. This is particularly clear in the BHI from all EEG bands to the HRV-HF frequency band in both subject groups and from the whole brain spectrum to the HRV-LF band only in the healthy controls group. It is interesting to note that very high values have been measured in both classes related to the BHI from the brain to the HRV-HF band.

**Fig. 4:**
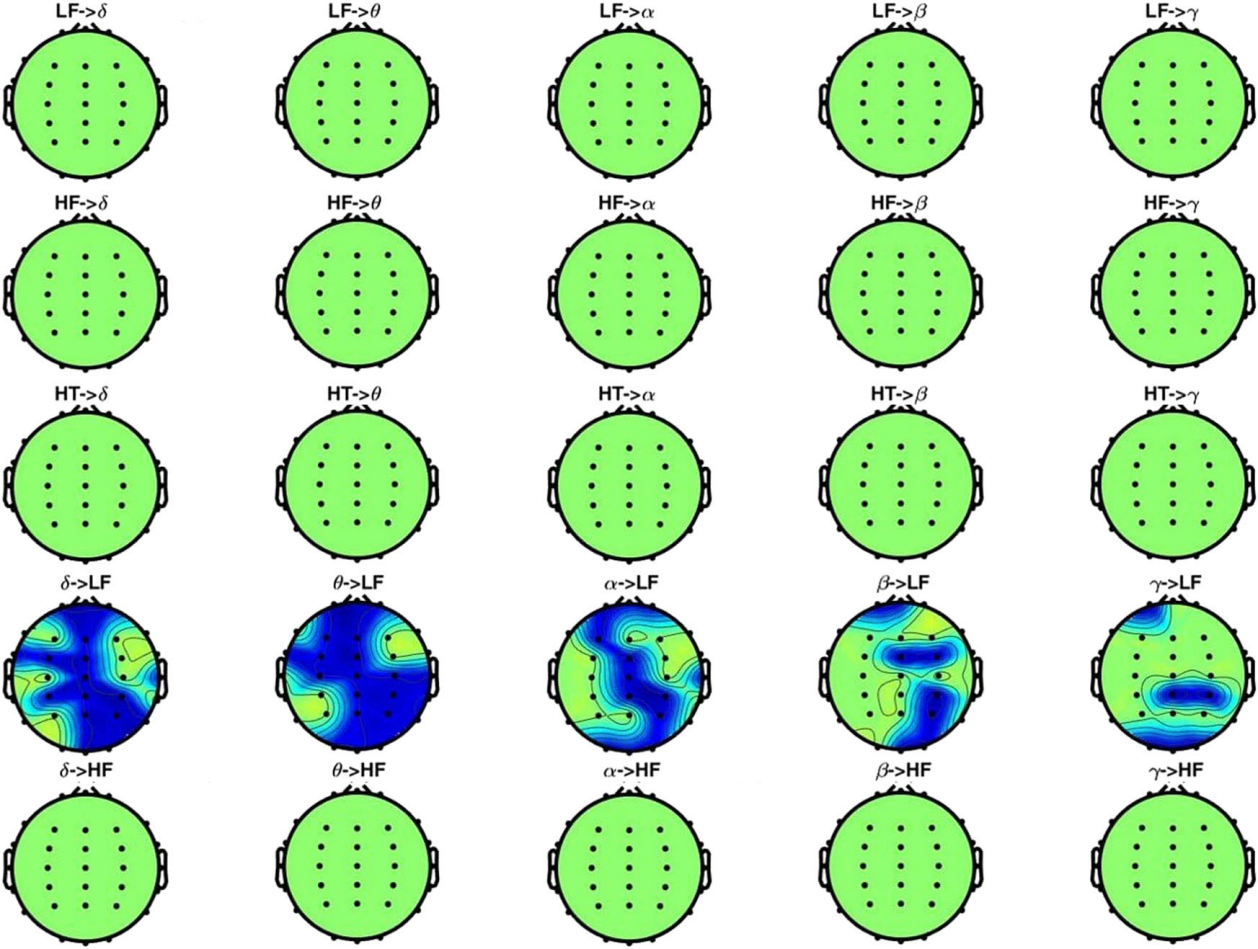
*P*-value topographic maps from non-parametric Mann–Whitney tests for unpaired samples in the proposed brain–heart model, between healthy subjects and dysphoric ones, in a resting state. Significant brain regions (*p* < 0.05, corrected through multiple comparisons) are highlighted with respect to the green areas, which indicate no significant changes between conditions. Blue regions represent a BHI that is significantly higher in dysphoric subjects.

Results from the statistical analysis of the SDG model output are depicted in Fig. 4, where the green areas indicate that changes between groups are not significant, whereas the blue areas indicate that the BHI values of the dysphoric subjects are higher than those of the control group. No red areas are highlighted, indicating that generally healthy subjects did not have higher BHI indexes than the dysphoric individuals. Figure 4 shows that all the combinations of heart-to-brain interactions (i.e. first three rows) and the BHI from the brain to the HRV-HF band (i.e. last row) are not significant.

The only significant maps in the figure are those belonging to the fourth row, showing the BHI from the brain to the HRV-LF band. The statistically significant regions are primarily located along the central vertical axis of the maps, with the EEG-γ band being less involved. More specifically, the between-groups from-brain-to-heart BHI indexes difference in the central region of the scalp was significant in almost all the EEG bands, together with the mid-frontal and occipital areas. The BHI indexes difference in prefrontal left and ventro-parietal right lobes appear to be more significant at lower frequencies (δ, θ, and α); however, the β band still depicts some significant electrodes in those regions. Particularly, the temporal lobes are progressively less spotlighted from the lower to higher frequency bands (i.e. from δ to γ), highlighting a much broader significant region in the δ and θ bands in comparison with the β and γ ranges.

**Fig. 5:**
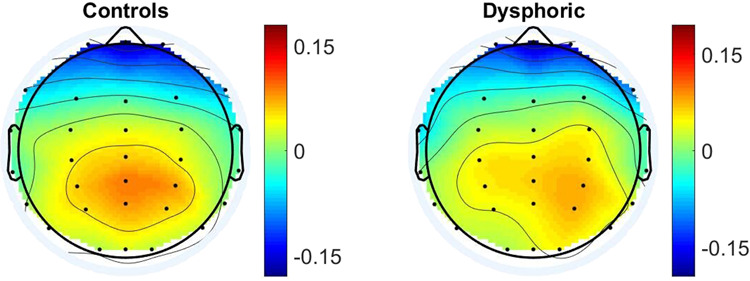
Topographic distribution of the extracted HEP indexes in healthy (left) and dysphoric (right) participants during 5 min in a resting state.

To investigate further the absence of significant heart-to-brain interplay, the HEP approach was applied; the experimental results are depicted in Fig. 5. The two topographic maps exhibit a high similarity, with higher HEP values in the posterior-central scalp areas and a negative gradient in the radial direction toward the external electrodes. A statistical analysis, performed on each channel using the non-parametric Mann–Whitney test for unpaired samples, did not enhance any channels to a 95% level of significance. Thus, HEP indexes that were extracted during a resting state in dysphoric subjects did not differ from healthy controls.

## Discussion

The present study investigated the functional directional BHI occurring in patients with dysphoria during a short-term resting state in 60 young females. Functional directional BHI was quantified using the SDG model^43^ on EEG and heartbeat oscillations. In particular, this work investigated functional coupling between EEG and sympathovagal dynamics and it identified a significantly higher from-brain-to-heart coupling in dysphoric subjects than in healthy controls. This occurred considering the HRV-LF, at the heartbeat level, and different frequency bands at the central level. The central axis between the hemispheres, from the midline frontal to occipital cortex, was the most activated.

More specifically, these results evidence that major changes in BHI occur at low EEG frequencies (i.e. δ, θ, and α), as opposed to higher frequencies (i.e. β and γ). EEG low frequency ranges have been associated both with cardiovascular control^69,70^, and with depressive symptoms^8–10^. In addition, EEG functional connectivity changes in the θ and α frequency bands were measured in healthy subjects when introspective mental rumination was performed^6^, which is a peculiar depressive/dysphoric symptom, and similar changes were obtained comparing healthy and dysphoric individuals^51^. Our results might give insights on the BHI role in the relationship bounding the EEG connectivity changes at low frequencies^6,51^, and the association found between rumination, other depressive symptoms, or meditation to sympathovagal dysfunctions^70–72^.

Furthermore, focusing on the BHI indexes from the EEG-α band to the HRV-LF band, depicted in Fig. 4, a region vertically spanning from the right posterior to the left anterior areas is highlighted. Intriguingly, similar patterns have already been pointed out during investigations of EEG asymmetries in clinical and subclinical depression in the α frequency band^7,9,10^. At a speculation level, this result may link the BHI phenomenon to the appetitive motivational system dysfunctions, suggesting further investigation in this field.

Note that a previous study on heartbeat dynamics in dysphoria showed significant differences at rest in the mean and standard deviation of RR series, as well as LF power with respect to healthy controls, whereas no differences between groups were observed in HF power or LF/HF ratio^29^. It could be argued that such changes in LF power may partially be due to an intensification of the cortical control onto sympathovagal dynamics through oscillations in the LF band.

The present study further investigated heart-to-brain interplay using the HEP approach and did not observe any significant difference between healthy controls and dysphoric subjects. Our results, which were obtained by applying the two methodologies (i.e. SDG and HEP), are consistent in depicting a comparable heart-to-brain interplay in the two experimental groups. Moreover, our results confirm the hypotheses of a higher CNS influence on heart dynamics in individuals with depressed mood^30^. These results suggest a possible involvement of CAN dysfunctions in dysphoria. While the interplay between subcortical and cortical CAN areas and its effect on EEG dynamics are currently unknown, our results suggest a dysfunctional activity of the MPFC in dysphoria. Moreover, differences between groups were also linked to posterior midline regions, which might result from the activity of posterior cingulate and the precuneus cortices. Further, these findings functionally mirror the causal relationship of over-activation in CAN-associated regions, such as the caudal subgenual region of primate ventro-medial prefrontal cortex, anticipating the cardiovascular and behavioural arousal typical of stress-related disorders^73^.

Possibly, an alteration of such control may partially explain the clinical interaction between depressed mood and cardiac disorders such as the increased risk of cardiovascular diseases (e.g. CHD) in patients diagnosed with mood disorders^30^ and vice versa^25–27^. Interestingly, a solo-EEG analysis that was recently performed on the same dataset^51^ did not identify any difference between the two experimental groups when investigating changes in power distributions, thus strengthening the necessity of the BHI approach to highlight psychophysiological patterns correlated with the psychopathology.

Owing to previous evidence linking depression to HRV abnormalities^16,31^, and linking depression to dysfunctional activity at the CAN level^14,74^, and considering the results presented in this work, it can be claimed that dysphoria may be associated with irregular functional BHI changes.

This work comes with limitations: larger experimental groups are needed and different physiological conditions than resting state remain to be investigated. Future studies should inspect the role of inter-subject variability and other brain- and heart-related physiological markers, owing to the known heterogeneity of mood disorders^75^.

## Conclusion

This study suggested that mood disorders, such as dysphoria, are associated with impaired functional BHI. Specifically, dysphoria is characterized by an overdrive of the functional neural control on heartbeat regulation, which acts through different brain oscillations to the sympathovagal dynamics and the so-called central-autonomic network at rest^76^. No significant differences were associated with the HRV-HF power, suggesting that the sympathetic nervous system and sympathovagal interplay are crucial in dysphoria.

In conclusion, the BHI approach constitutes a promising framework to yield biomarkers for mood disorders, with the aim to develop an objective characterization and diagnosis of mood disorders.
